# Identification of hub genes significantly linked to subarachnoid hemorrhage and epilepsy *via* bioinformatics analysis

**DOI:** 10.3389/fneur.2023.1061860

**Published:** 2023-01-19

**Authors:** Hong Gao, Jie Li, Qiuping Li, Yuanxiang Lin

**Affiliations:** ^1^Department of Neurosurgery, The First Affiliated Hospital, Fujian Medical University, Fuzhou, Fujian, China; ^2^Department of Neurosurgery, Zhongshan Hospital (Xiamen), Fudan University, Xiamen, Fujian, China; ^3^Department of Medical Intensive Care Unit, Tongji Medical College, Maternal and Child Health Hospital of Hubei Province, Hua Zhong University of Science and Technology, Wuhan, Hubei, China

**Keywords:** subarachnoid hemorrhage (SAH), epilepsy, immune-related genes (IRGs), MMP9, C3AR1

## Abstract

**Background:**

Although epilepsy has been linked to subarachnoid hemorrhage (SAH), the underlying mechanism has not been fully elucidated. This study aimed to further explore the potential mechanisms in epilepsy and SAH through genes.

**Methods:**

Gene expression profiles for subarachnoid hemorrhage (GSE36791) and epilepsy (GSE143272) were downloaded from the Gene Expression Omnibus (GEO) database. Differential expression analysis was performed to identify the common differentially expressed genes (DEGs) to epilepsy and SAH, which were further analyzed by functional enrichment analysis. Single-sample gene set enrichment analysis (ssGSEA) and weighted correlation network analysis (WGCNA) were used to identify common module genes related to the infiltration of immune cells in epilepsy and SAH. Hub module genes were identified using a protein–protein interaction (PPI) network. Finally, the most relevant genes were obtained by taking the intersection points between the DEGs and hub module genes. We performed validation by retrospectively analyzing the RT-PCR levels of the most relevant genes in patients with pure SAH and patients with SAH complicated with epilepsy. Our experiments verified that the SAH and SAH+epilepsy groups were significantly different from the normal control group. In addition, significant differences were observed between the SAH and SAH+epilepsy groups.

**Results:**

In total, 159 common DEGs–85 downregulated genes and 74 upregulated genes—were identified. Functional analysis emphasized that the immune response was a common feature to epilepsy and SAH. The results of ssGSEA and WGCNA revealed changes in immunocyte recruitment and the related module genes. Finally, MMP9 and C3aR1 were identified as hub genes, and RT-PCR confirmed that the expression levels of the hub genes were higher in epilepsy and SAH samples than in normal samples.

**Conclusions:**

Our study revealed the pathogenesis of SAH complicated with epilepsy and identified hub genes that might provide new ideas for further mechanistic studies.

## Introduction

Epilepsy, as a manifestation of brain injury, is caused, on the one hand, by inflammatory reactions, tumors, cerebrovascular diseases, trauma, and other brain diseases or injuries and, on the other, by iatrogenic injuries such as surgical interventions. Epilepsy caused by subarachnoid hemorrhage (SAH) mostly originates from aneurysm rupture, which can occur at the onset of SAH, before or after surgery (1). With current treatments, complications still occur in nearly 50% of SAH survivors. Epilepsy is one such complication that gravely affects patients' quality of life (2). The excessive mortality rate after the diagnosis of symptomatic epilepsy is well known and is related to the epilepsy itself, its treatment, accidents and suicides, sudden unexpected death in epilepsy, and status epilepticus. Moreover, SAH combined with epilepsy leads to anxiety, depression, and other psychological problems, which may be worse than primary disease. However, the potential mechanisms of epilepsy secondary to SAH are still unclear (3–5).

Inflammation may be an important factor in epilepsy induced by SAH, as confirmed in previous studies (6). Multiple studies have also shown that hyperactivation of the immune system in epilepsy patients further contributes to the high incidence of seizures in patients with autoimmune diseases (7, 8). Only a few studies have explored the genetic relationship between cerebral hemorrhage and epilepsy, and there is a lack of genetic studies concerning the relationship between SAH and epilepsy (9, 10).

We hypothesized that there might be some hub gene relationship between SAH and epilepsy. In this study, we investigated the underlying mechanism of SAH-induced epilepsy by finding common molecular changes in the two diseases. First, we performed differential expression analysis of the gene expression profiles of subarachnoid hemorrhage (GSE36791) and epilepsy (GSE143272) using single-sample gene set enrichment analysis (ssGSEA) and weighted correlation network analysis (WGCNA), among other methods, to finally elucidate the most relevant genes. Clinically significant genes were identified and their expression levels were then validated in patients with epilepsy and SAH by quantitative RT-PCR.

## Methods

### Data collection

**Figure 1:**
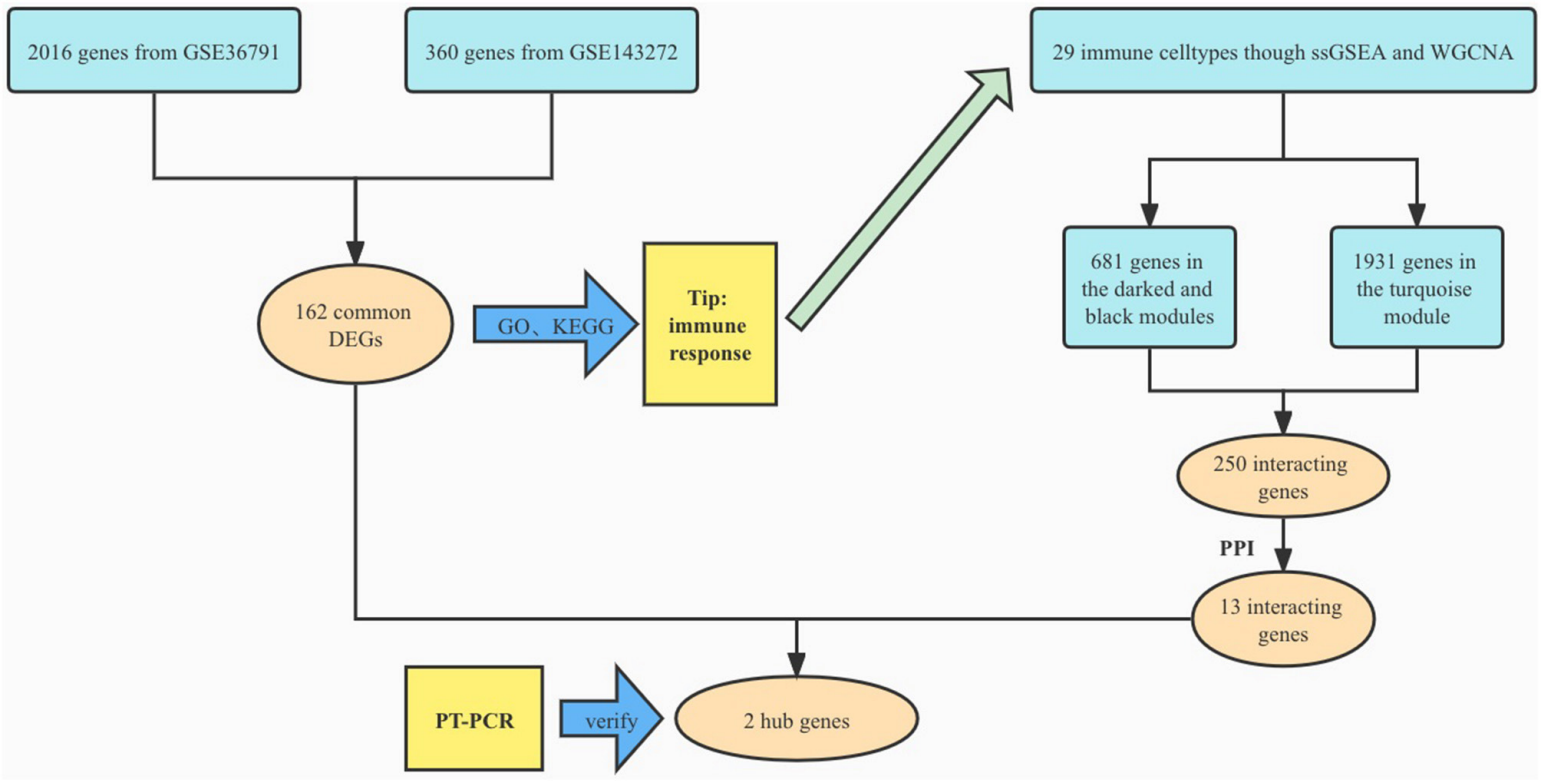
Study flowchart.

We searched the Gene Expression Omnibus (GEO) database for aneurysmal SAH and epilepsy gene expression profiles. The following criteria were applied to filter the dataset: (1) the gene expression profiling must include cases and controls; (2) the organization used for sequencing should be peripheral blood mononuclear cell; (3) the number of samples in each group should not be <10 to ensure the accuracy of the WGCNA; (4) these datasets must provide the processed data or raw data that could be used for re-analysis; and (5) the GEO datasets GSE143272 and GSE36791 were selected. We then performed log2 transformation of the gene expression profiling and matched the probes to their gene symbols according to the annotation document of the corresponding platforms. Finally, a gene matrix with row names as sample names and column names as gene symbols was obtained for subsequent analyses (Figure 1).

### Identification of differentially expressed genes

Differentially expressed gene (DEG) analysis was conducted in the datasets GSE143272 and GSE36791 using the R package limma. Genes with an adjusted *P* < 0.05 and |fold change (FC)| > 1.3 were considered DEGs. The volcano map of DEGs was plotted in R software while the online Venn diagram tool was used to obtain the common DEGs.

### Gene ontology and pathway enrichment analysis

Gene Ontology (GO) terms and Kyoto Encyclopedia of Genes and Genomes (KEGG) pathway enrichment analysis were performed by using clusterProfiler. The terms obtained from the KEGG pathway and GO analyses (including molecular functions, biological processes, and cellular components) that had false discovery rates (FDRs) < 0.05 were considered to be significantly enriched in these shared DEGs.

### Evaluation of immune cell subtype distribution

The relative abundances of immune cells were evaluated with ssGSEA using the R package GSVA. ssGSEA applies the genetic characteristics of immune cell populations to individual cancer samples (11, 12). The infiltration scores of 28 immune cells were calculated using a deconvolution algorithm.

### Weighted gene co-expression network analysis

The R package WGCNA was used to construct a weighted co-expression network based on the expression values of 4,561 genes. First, the proper soft-thresholding power β was chosen based on the criterion of approximate scale-free topology. The function pickSoftThreshold was used to analyze network topology and calculate the soft-thresholding power β. The network was constructed with the automatic network construction function. Second, according to the dissimilarity coefficient between genes, a hierarchical clustering tree was built for module detection. Gene significance and module membership were defined to quantify the correlation between modules and clinical characteristics. Ranked by the absolute value of module significance, modules showing a strong association with the specific cell subtypes were selected as hub modules.

### Protein–protein interaction network construction and module analysis

Search Tool for the Retrieval of Interacting Genes (STRING; http://string-db.org/) (version 11.0) can search for the relationship between proteins of interest, such as direct binding relationships or coexisting upstream and downstream regulatory pathways, to construct a protein–protein interaction (PPI) network with complex regulatory relationships. Interactions with a combined score exceeding 0.4 were considered statistically significant. Cytoscape (http://www.cytoscape.org/) (version 3.7.2) was used to visualize this PPI network. The Cytoscape plug-in MCODE (Molecular Complex Detection) was used to analyze key functional modules. The selection criteria were set as: K-core = 2, degree cutoff = 2, max depth = 100, and node score cutoff = 0.2.

### Selection and analysis of hub genes

Hub genes were identified by using the cytoHubba plug-in of Cytoscape. Here, we applied seven common algorithms—MCC, MNC, Degree, Closeness, Radiality, Stress, EPC—to evaluate and select hub genes. We then constructed a co-expression network of these hub genes *via* GeneMANIA (http://www.genemania.org/), which is a reliable tool for identifying internal associations in gene sets.

### Clinical information

With the approval of our institutional ethics review board, we collected clinical information from 21 persons (Table 1), 13 of whom had been diagnosed with SAH following digital subtraction angiography. The clinical data were collected between June 2021 and August 2022 from the Department of Neurosurgery in Zhongshan Hospital (Xiamen), Fudan University, in China.

**Table 1:** The clinical characteristics of 21 participants.

**Groups**	**Normal**	**SAH**	**SAH + epilepsy**
Ages (Y)	54.6 ± 14.1	57.1 ± 9.8	56.6 ± 17.7
Time of symptoms (H)	0	2.8 ± 1.2	2.8 ± 1.1
Hypertension (N)	5	5	6
Diabetes (N)	3	3	3
Smoking (N)	5	5	4
Drinking(N)	7	3	4
Family history (N)	0	0	0

SAH, subarachnoid hemorrhage; Y, years; H, hours; N, numbers.

### RNA extraction and quantitative RT-PCR

TriPure samples were added with lysis buffer to cells and they were completely lysed by pipetting several times. After chloroform was added, the samples were shaken vigorously for 15 s and left at room temperature for 3 min. The samples were then centrifuged at 12,000 × *g* for 15 min at 4°C. The aqueous phase was next transferred to a new tube. The RNA was precipitated by the addition of 0.5 mL of isopropanol to each 1 mL of TriPure and an incubation at room temperature for 10 min. After centrifugation at 12,000 × *g* for 10 min at 4°C, gelatinous precipitates were evident on the side and bottom of the tube. The RNA pellet was washed with 75% ethanol and the supernatant was discarded. After the samples were left to dry at room temperature, 50 μL of DEPC water was added for reverse transcription. RT-qPCR was performed on the cDNA obtained by reverse transcription (the internal reference gene was GAPDH) (Table 2).

**Table 2:** Primer design.

**Primer name**	**Primer sequence**
hC3AR1 qRT F	GAAACCAGCCCACTGGATAA
hC3AR1 qRT R	TGTTGGCACTTGATCGTCAT
hMMP9 qRT F	TTGACAGCGACAAGAAGTGG
hMMP9 qRT R	TCACGTCGTCCTTATGCAAG
hGAPDH F	CAAGGTCATCCATGACAACTTTG
hGAPDH R	GTCCACCACCCTGTTGCTGTAG

### Statistical analysis

Experimental qRT-PCR data are presented as the mean ± standard error of the mean (SEM). SPSS 23.0 was used to perform statistical analyses. Differences between experimental groups were determined by applying unpaired *t*-tests.

## Results

### Identification of DEGs

**Figure 2:**
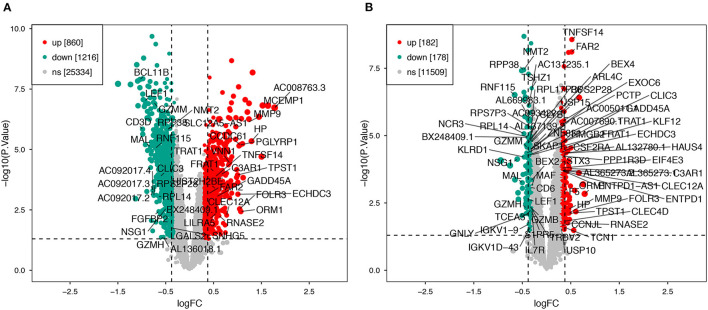
Volcano plot of DEGs. **(A)** Extraction of related DEGs in SAH. **(B)** Extraction of related DEGs in epilepsy. Green and red represent downregulated and upregulated genes, respectively. SAH, subarachnoid hemorrhage.

**Figure 3:**
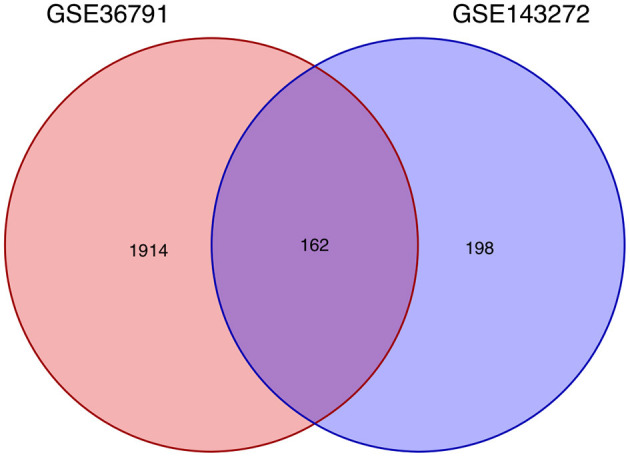
Intersecting genes of GSE36791 and GSE143272. Red and purple represent GSE36791 and GSE143272, respectively.

Using the previously established criteria, DEGs (GSE36791 and GSE143272) were identified (Figure 2). To explore the mechanisms underlying the two diseases, we searched for DEGs in SAH and epilepsy. SAH had 800 upregulated genes and 1,216 downregulated genes, whereas epilepsy had 182 upregulated genes and 178 downregulated genes. The intersection of the Venn diagram obtained 162 common DEGs (Figure 3).

### Gene ontology and pathway enrichment analysis

**Figure 4:**
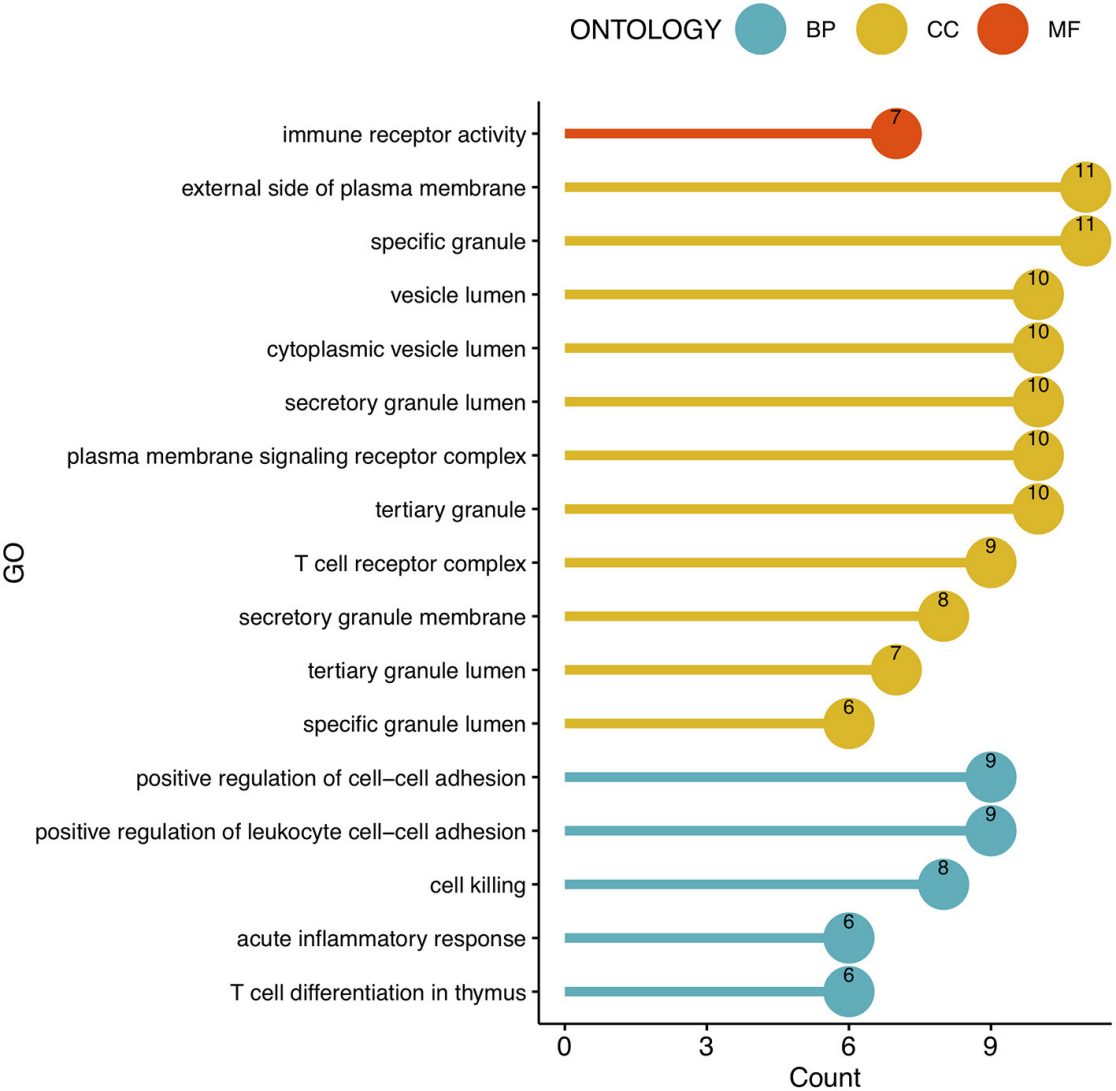
Bubble diagram of GO enrichment functions. Green, yellow, and red dots represent counts. GO, Gene Ontology; BP, Biological Process; CC, Cell Component; MF, Molecular Function.

**Figure 5:**
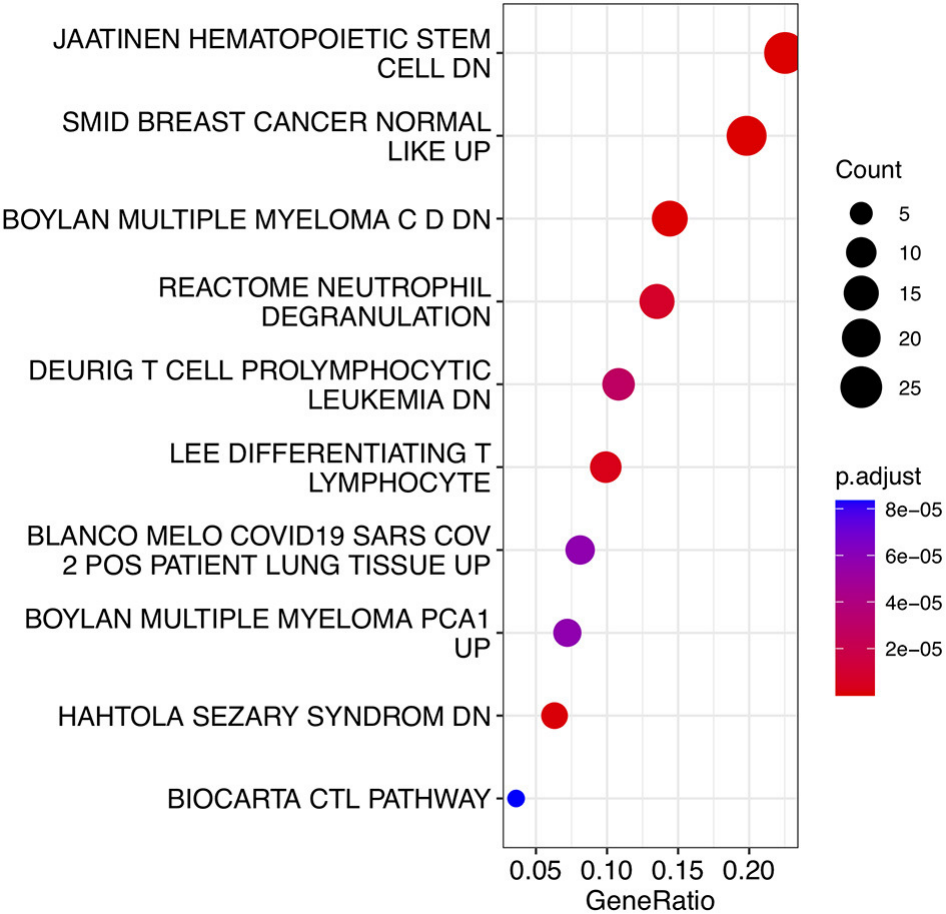
Bubble diagram of KEGG pathways. Red and blue dots represent P values. KEGG, Kyoto Encyclopedia of Genes and Genomes.

To analyze the biological functions and pathways related to the 162 common DEGs, GO and KEGG pathway enrichment analyses were performed. GO analysis revealed the involvement of parts of several important pathways, such as immune receptor activity, T cell receptor complex, positive regulation of cell-cell adhesion, positive regulation of leukocyte cell-cell adhesion, cell killing, acute inflammatory response, and T cell differentiation in thymus (Figure 4). In terms of KEGG analysis, parts of several important pathways were involved, such as REACTOME NEUTROPHIL DEGRANULATION, DEURIG T CELL PROLYMPHOCYTIC LEUKEMIA DN, and LEE DIFFERENTIATING T LYMPHOCYTE (Figure 5). These results strongly suggested that the immune response was associated with SAH and epilepsy.

### Evaluation of immune cell subtype distribution

**Figure 6:**
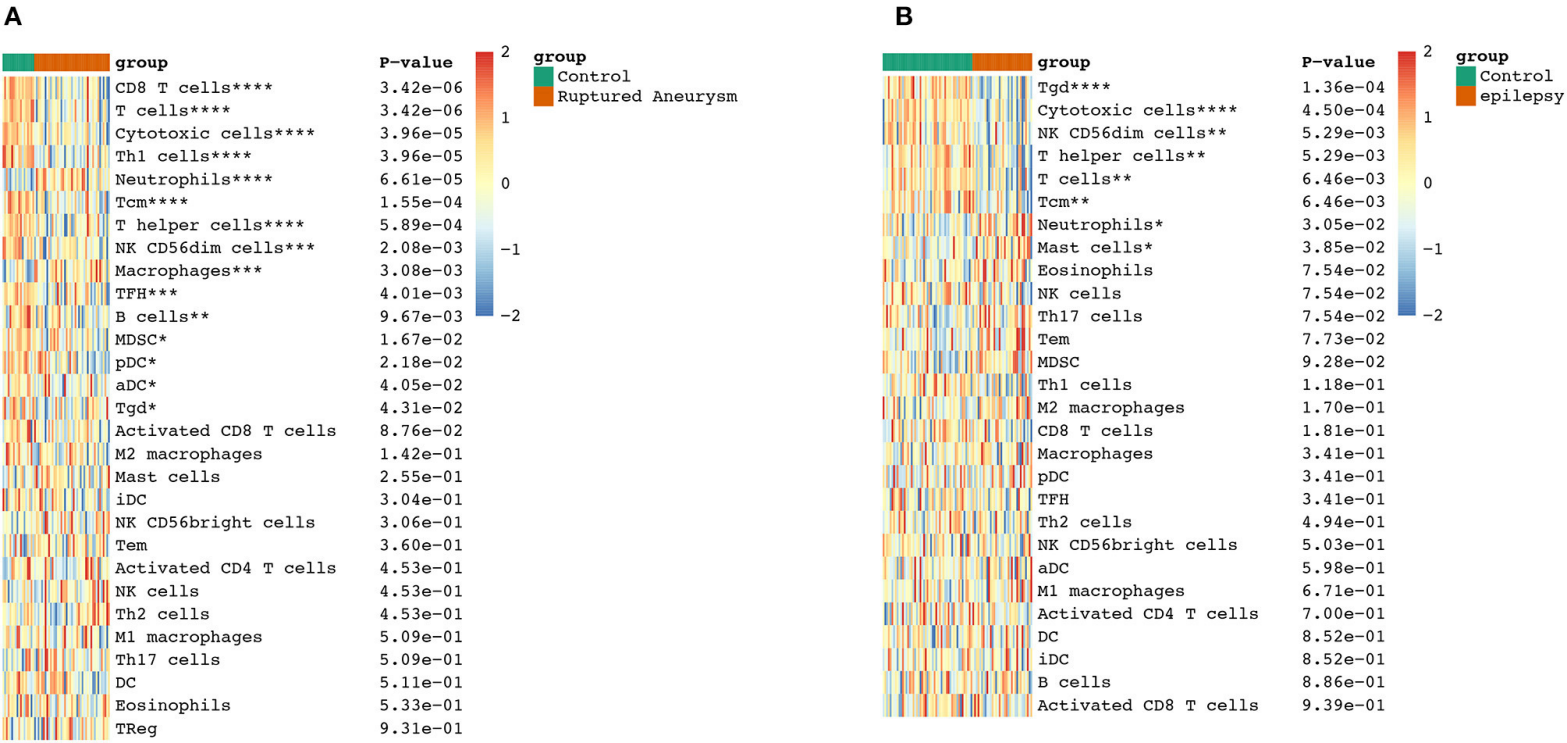
Extraction of immune-related DEGs in SAH **(A)** and epilepsy **(B)**. Green and red represent the control and experimental groups, respectively. DEGs, differentially expressed genes; SAH, subarachnoid hemorrhage. *P < 0.05; **P < 0.01; ***P < 0.005; ****P < 0.001.

To further explore the correlation between the immune cell and immune response in the two diseases, we used the ssGSEA algorithm to evaluate the immune infiltration of 29 immune cell types. The results showed that the SAH group was associated with a higher proportion of CD8 T cells, T cells, cytotoxic cells, Th1 cells, neutrophils, T central memory (TCM) cells, T helper cells, natural killer (NK) CD56^dim^ cells, macrophages, T follicular helper cells, B cells, myeloid-derived suppressor cells, plasmacytoid dendritic cells, activated dendritic cells (aDC), and T gamma delta (Tgd) cells. We also observed a correlation of Tgd cells, cytotoxic cells, NK CD56^dim^ cells, T helper cells, T cells, TCM cells, neutrophils, and mast cells with epilepsy. The distribution of the intensity values for the normalized expression data from all of the SAH and epilepsy samples is shown in the Heatmap plot of Figure 5. Finally, the results indicated that T cells, cytotoxic cells, Tgd cells, NK CD56^dim^ cells, T helper cells, TCM cells, and neutrophils were the common infiltrating immune cells in SAH and epilepsy (Figure 6).

### Weighted gene co-expression network analysis

**Figure 7:**
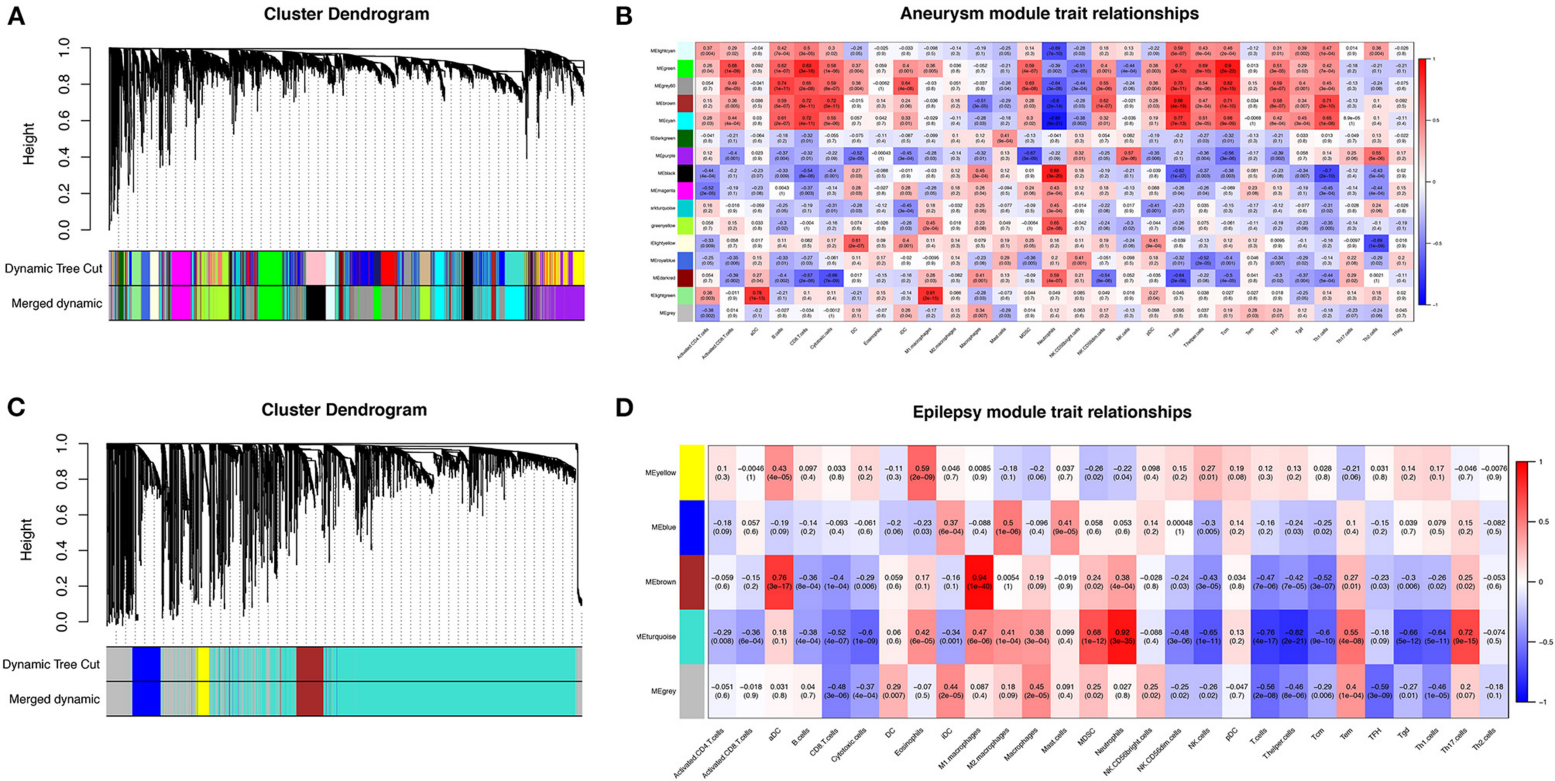
Weighted gene co-expression network analysis (WGCNA). **(A)** Cluster dendrogram of co-expression genes in SAH. **(B)** Module–trait relationships in SAH. Each cell contains the corresponding correlation and P value. **(C)** Cluster dendrogram of co-expression genes in epilepsy. **(D)** Module–trait relationships in epilepsy. Each cell contains the corresponding correlation and P value. SAH, subarachnoid hemorrhage.

To further search for hub genes related to the above immune cells, the fraction of immune cell infiltration was additionally processed with the WGCNA package in R software. These modules which were identified and constructed were represented with different colors (Figures 7A, C). The main findings of the module–trait correlation analyses are shown in Figures 7B, D (each cell contains the correlation coefficient and corresponding *P* value). Combined with the co-immune cells obtained from ssGSEA, Figure 7 summarizes the significance of the immune cell types related to each module. The results indicated that the dark red and black modules were clearly related to the above immune cells in SAH (Figure 7B). Thus, the dark red and black modules, which included 681 genes, were considered to be a hub module correlated with the infiltrating immune cells. The results also indicated that the turquoise module was clearly related to the above immune cells in epilepsy (Figure 7D). Thus, the turquoise module, which included 1,931 genes, was considered a hub module correlated with the infiltrating immune cells.

**Figure 8:**
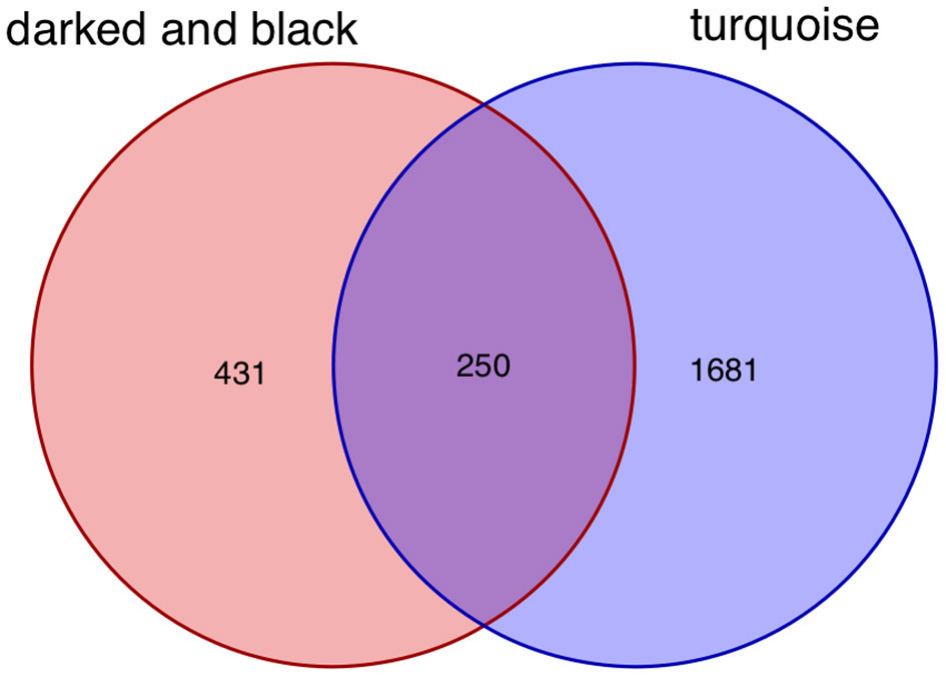
Intersecting genes of the dark red and black modules and the turquoise module. Red represents the dark red and black modules, whereas purple represents the turquoise module.

After taking the intersection of the Venn diagram for dark red and black in the aneurysm module and turquoise in the epilepsy module, 250 interacting genes were obtained (Figure 8).

### PPI network construction and DEGs

**Figure 9:**
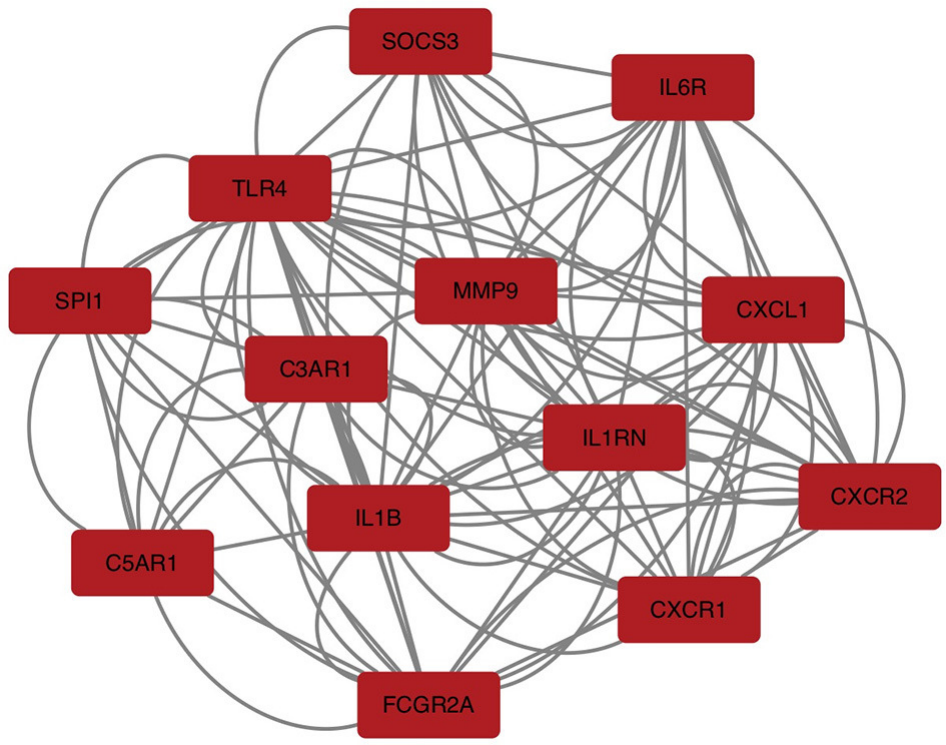
PPI network of hub module genes related to the infiltration of immune cells. The closer the relationship, the higher the number of adjacent nodes of genes. PPI, protein–protein interaction.

To explore the relationship of the module hub genes of the above 250 interacting genes for SAH and epilepsy, we used the STRING database to construct a PPI network with a combined score >8.833. This network contained 13 nodes and 106 edges, and the top 13 interacting genes were used in further analysis (Figure 9).

**Figure 10:**
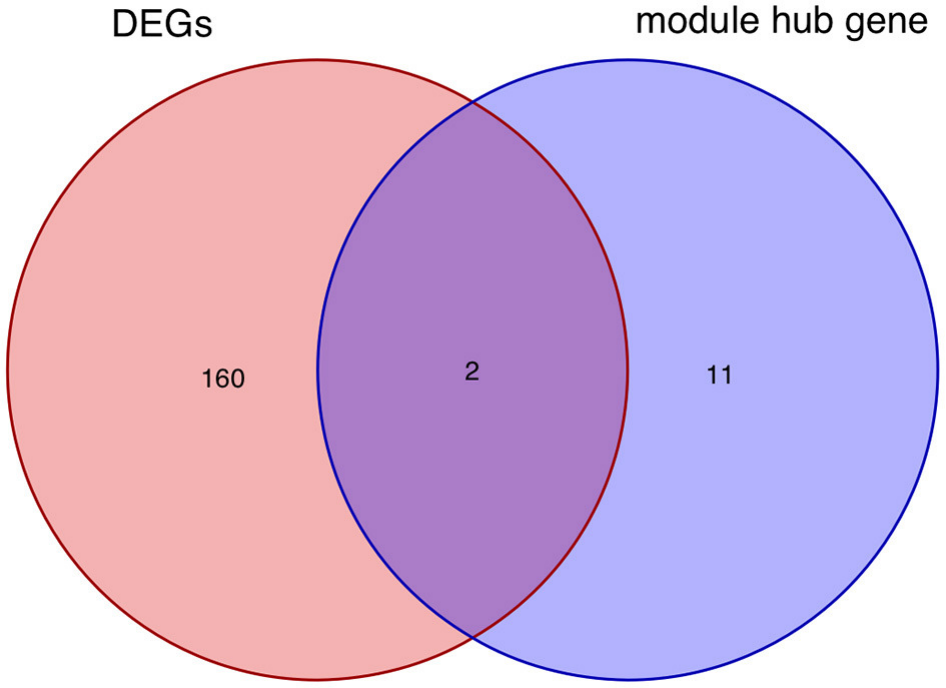
Intersecting genes of DEGs and module hub genes. Red and purple represent DEGs and module hub genes, respectively. DEGs, differentially expressed genes.

After taking the intersection of the Venn diagram for DEGs and module hub genes, the results identified the two interacting genes to be MMP9 and C3AR1 (Figure 10).

### RNA extraction and quantitative RT-PCR

**Figure 11:**
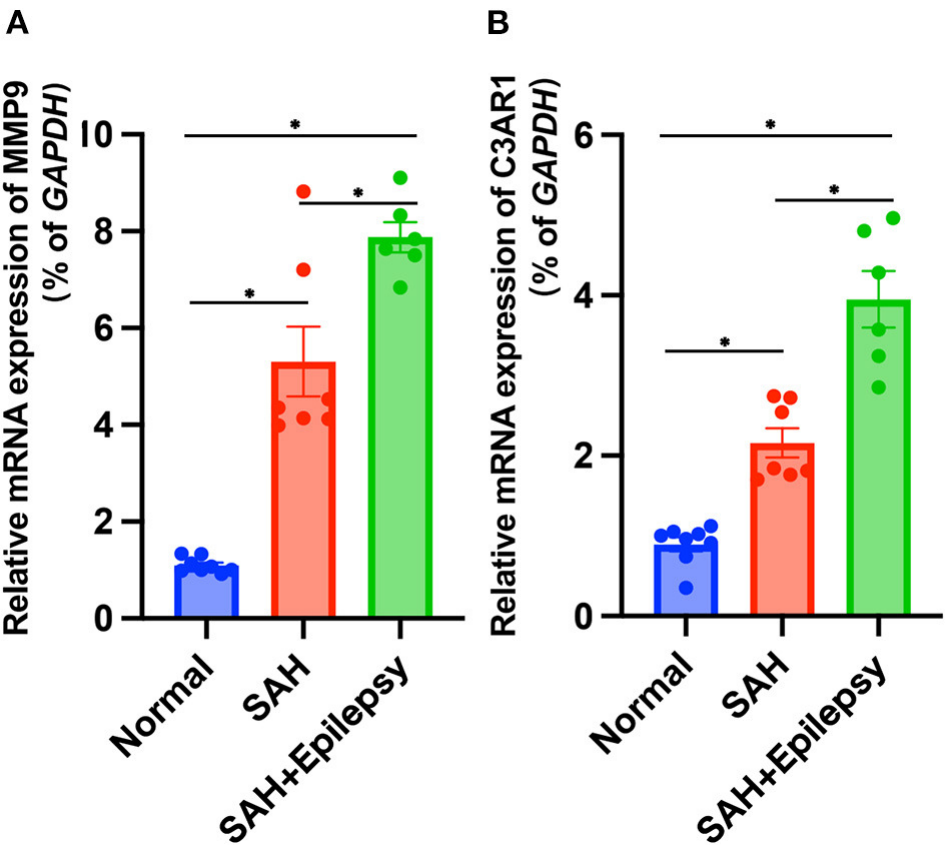
Validation of RT-qPCR levels *via* patients. Blue, red, and green represent normal, SAH, and SAH + epilepsy, respectively. SAH, subarachnoid hemorrhage. *P < 0.05.

The bioinformatics analysis results from the GSE36791 and GSE143272 datasets indicated that two genes—MMP9 and C3AR1—were strongly associated with SAH complicated with epilepsy (Figure 11). The SAH and SAH+epilepsy groups were significantly different from the normal control group (*P* < 0.05). In addition, there were significant differences between the SAH and SAH+epilepsy groups (*P* < 0.05).

## Discussion

As a severe complication of SAH, epilepsy increases mortality and disability rates (13). Although the underlying mechanisms of epilepsy after SAH remain unclear, several classes of risk factors have been reported to contribute to a greater risk of epilepsy after SAH, such as hemosiderin deposition, subarachnoid clot burden, hypertension, vasospasm, and acute hydrocephalus (3, 14, 15). Hemosiderin deposition in the brain and spinal cord due to hemorrhage into the subarachnoid space in some patients is considered to be a cause and key predictor of epilepsy after SAH (16). Only a few genetic studies have been conducted on epilepsy and cerebrovascular disease, and fewer on SAH (17, 18). The goal of our study was to elucidate the hub genes linking SAH and epilepsy, which may contribute to the optimization of future epilepsy strategies.

Based on these factors, we conducted this study to identify key significant DEGs among SAH, epilepsy, and normal samples and to explore the biological functions and expression levels of the genes. First, we took the intersection of two initial DEGs to obtain 162 DEGs related to SAH and epilepsy. GO and KEGG were performed on the intersecting DEGs and analysis revealed that most of these genes were related to immune regulation. Therefore, we next used the ssGSEA and WGCNA methods to analyze the cell types of the SAH and epilepsy samples. The intersecting genes were screened to obtain 13 module hub genes through the PPI network. Finally, we detected two genes—MMP9 and C3AR1—*via* the overlap of the 162 related genes and 13 module hub genes, and further verified their gene expression levels using RT-PCR. Thus, SAH may induce epilepsy through the regulation of pathogenic pathways by immune-related genes (IRGs), such as the expression levels of the MMP9 and C3AR1 genes in the immunoregulatory pathways leading to the occurrence of epilepsy.

The roles of MMP-9 and C3AR1 in the pathogenesis of epilepsy have been reported previously, but the mechanisms of the genes associated with SAH-induced epilepsy had not been studied (19, 20). The MMP9 gene exerts profound effects on epilepsy by regulating immune cell infiltration and inflammation (21). C3AR1 acts as a protein-coding gene in which C3a is a pro-inflammatory mediator released during complement system activation. In the present study, higher MMP-9 and C3AR1 levels in SAH were associated with the incidence of epilepsy after SAH. Many SAH patients had elevated levels of MMP-9 and C3AR1 in our study, which may be directly related to the physiological inflammation and repair processes that occur in the hemorrhagic area and surrounding brain structures. Studies have shown that elevated levels of MMP-9 can lead to blood–brain barrier disruption and SAH leukocyte entry into the central nervous system, thereby exacerbating inflammation and subsequent brain damage (22). When MMP-9 levels exceed physiological repair thresholds, SAH-induced reorganization is excessive, leading to irreversible changes and abnormal rewiring (23). Severe SAH may cause cells to release MMP-9, and high levels of MMP-9 were detected in human blood in our study. High MMP-9 levels are strongly associated with worse clinical outcomes, including longer intensive care unit stays and a higher risk of death (24). C3aR1 is a key regulator of neuroinflammation pathogenesis and mediates immune networks in the central nervous system, because its direct target is STAT3 (signal transducer and activator of transcription). Studies have shown that C3aR1 promotes neuroinflammation, synaptic defects, and neurodegeneration in mice (25). A large number of studies have also shown that C3aR1 mediates the increase in the expression of pro-inflammatory factors such as IL-1, IL-6, and TNF-α in the acute phase of SAH (16). In conjunction with neutrophil infiltration, the blood–brain barrier will be destroyed, brain edema will form, and heme metabolism will be affected, thereby aggravating the pro-inflammatory response. Neuroinflammation is closely related to the occurrence of epilepsy (26), which also indicates that increased MMP-9 and C3AR1 content may have a synergistic effect on the occurrence of epilepsy through inflammation. The higher level of hub genes may be related to the extent of blood–brain barrier destruction and brain injury. These are the bases for the occurrence of epilepsy, but the relationship between SAH-induced changes in the expression of IRGs and epilepsy requires further study.

Previous studies have separately explored the related immune genes of SAH and epilepsy (27, 28). However, few studies have explored their shared molecular mechanisms through advanced bioinformatics methods. Due to the high prevalence of SAH complicated with epilepsy, we investigated and identified the common DEGs of SAH and epilepsy for the first time, which will help to further elucidate the pathogenesis of SAH complicated with epilepsy. We found 162 overlapping DEGs in both conditions, and GO and KEGG pathway enrichment analysis revealed that these genes were significantly enriched in inflammatory and immune pathways, such as the T cell receptor complex. We also found T cells, cytotoxic cells, Tgd cells, NK CD56^dim^ cells, T helper cells, TCM cells, and neutrophils to be the main infiltrating immune cells in ssGSEA. WGCNA demonstrated that immune cells play an important role in both diseases. The hub genes that were finally discovered were the IRGs MMP9 and C3AR1. However, a limitation of our study is that it was a retrospective study, and considerable external validation is required to validate our findings. It is necessary to further explore the gene functions of MMP9 and C3AR1, which might be helpful for the treatment of such diseases.

Over the past few decades, inflammation and immune responses have been suspected to be involved in one of the underlying mechanisms of epilepsy, and a series of targeted therapies have subsequently been derived (29, 30). Once the hub genes of the disease are identified, targeted therapies can be considered. Because severe SAH can lead to the occurrence of epilepsy, SAH may influence immune cell infiltration and signaling pathway transduction through IRGs, thereby affecting the development of epilepsy episodes (31). Indeed, MMP9 and C3AR1 are IRGs. Unfortunately, few studies have investigated the relationship among IRGs, SAH, and epilepsy. We constructed a complex interaction network to identify key nodes through their common DEGs. This comprehensive bioinformatics approach has been shown to be reliable in a variety of diseases (32, 33). Here, we investigated differences in immune cell infiltration among normal samples, SAH samples, and SAH+epilepsy samples. The results showed that the expression of MMP9 and C3AR1 was higher in SAH patients than in normal individuals and that the incidence of epilepsy in SAH patients with significant expression of these two IRGs was significantly higher. Thus, the current work has proved that the detection of MMP9 and C3AR1 in severe SAH patients can help to predict the incidence of epilepsy and guide the prevention of epilepsy in the process of diagnosis and treatment.

## Conclusions

In summary, we identified the hub genes, namely, MMP9 and C3AR1, of SAH complicated with epilepsy and determined that they may work by regulating immune cell infiltration. However, the mechanism underlying the hub genes and immune cells in epilepsy due to SAH remains to be elucidated. This study provides new insights for the further study of the mechanism of epilepsy due to SAH.

## Data availability statement

The original contributions presented in the study are included in the article/supplementary material, further inquiries can be directed to the corresponding author.

## Ethics statement

The studies involving human participants were reviewed and approved by the Ethics Committee of Zhongshan Hospital, Fudan University (Xiamen). The patients/participants provided their written informed consent to participate in this study.

## Author contributions

YL, QL, and HG contributed to conception and design of the study. JL organized the database and performed the statistical analysis. HG wrote the first draft of the manuscript. JL and YL wrote sections of the manuscript. YL is responsible for ensuring that the submission adheres to all journal requirements. All authors contributed to manuscript revision, read, and approved the submitted version.
